# The Convalescent Mental Patient

**Published:** 1924-04

**Authors:** 


### THE CONVALESCENT MENTAL PATIENT.

The annual meeting of the Mental After Care Association, a society which is rapidly growing in influence and importance, was held on March 11 at the Mercers' Hall.

### The Appeal to the Imagination.

Sir Charles Wakefield, who presided, said that there had been a large increase in the number of cases helped and a substantial increase in the amount refunded by patients and their friends. The main difficulty, however, in securing an increase in our subscriptions (said Sir Charles) is that the appeal to the imagination—so necessary in all cases, and so simple and successful in pleading for, say, work amongst children—is not easy to frame in the case of mental after-care. The little orphaned child, the helpless invalid, the aged and needy—their plight touches at once the sources of sympathy in us all. The position of the man or woman who comes straight from the darkness of mental illness into an alien, indifferent and even seemingly hostile world is every whit as painful. If such help as we can offer is not available, there is appalling risk of a relapse. Naturally enough, the general public do not at first understand this peril of the released mental patient. They do not feel his shyness, his self-distrust, his strange baseless forebodings, and his possible loss of skill or physical health. Nor do they fully realise what a difference it makes if he can be shielded for a brief period from the first buffetings of our bluff but well-meaning world.

### "Phenomenal Ignorance."

Sir Humphrey Rolleston pointed out that the Association was by its action a notable example of the highest aim of medicine—not merely to cure disease but to prevent it. By averting the relapse into insanity of those it helped, the Society assisted in raising the whole standard of National Health. In a vigorous speech Sir James Crichton-Browne remarked that the ignorance of the public upon lunacy questions was phenomenal. During the past year the Mental After Care Association had afforded relief to 1,042 patients. All of these had given excellent testimony as to their treatment in various asylums and stated their readiness to return should the need arise. This testimony should go far to allay the wild and reckless statements which were being made in regard to these institutions. In view of the numerous difficulties with which they had to contend, he was convinced that no public institutions in this country were more efficiently and humanely conducted. It was true that some asylums were too large, some needed more of the hospital character imparted to them, and they should be more open to visitation by the kind-hearted. No one would welcome the Royal Commission more than the Medical Officers. He hoped that we should have an amendment of the lunacy law which would give adequate security to the patients, the public and medical men.

---

**A bibliography of Industrial Hygiene, which may be found useful for health workers, is published by the International Labour Office Bureau, 26 Buckingham Gate, S.W. 1.**

